# Superficial Type of Multiple Basal Cell Carcinomas: Detailed Comparative Study of Its Dermoscopic and Histopathological Findings

**DOI:** 10.1155/2011/385465

**Published:** 2010-10-04

**Authors:** Akiko Hirofuji, Kojiro Takiguchi, Koichiro Nakamura, Akira Kuramochi, Tetsuya Tsuchida, Eiichi Arai, Michio Shimizu

**Affiliations:** ^1^Department of Dermatology, Saitama Medical University, 1397-1 Yamane, Hidaka city, Saitama 350-1298, Japan; ^2^Department of Pathology, Saitama Medical University, 1397-1 Yamane, Hidaka city, Saitama 350-1298, Japan; ^3^Department of Pathology, Saitama International Medical Center, Saitama Medical University, 1397-1 Yamane, Hidaka city, Saitama 350-1298, Japan

## Abstract

We investigated in detail the dermoscopic and histopathological findings in a case of a superficial type of multiple basal cell carcinomas (BCCs). These multiple lesions (occurring in the chest, neck, and back) showed three different findings, respectively. Dermoscopy of the erythematous and brown-colored patch on the anterior chest showed spoke wheel areas, and the histopathological cross-section revealed vertical spoke wheel structures. In the black- and brown-colored patch at the neck, the dermatoscopy showed a maple leaf-like structure, which was in accordance with the strengthening of the histological lateral connection of the lesion. The brown-colored patch of the lateral back histologically showed irregularly enlarged spoke wheel-like areas with peripheral increased melanin pigments, which correlated with the dark black color of dermoscopic maple leaf-like areas. The vertical spoke wheel areas by dermatoscopy revealed a horizontal spoke wheel structure by histopathology.

## 1. Introduction

Although the gross findings noted in basal cell carcinomas (BCCs) have been quite varied, there are several common diagnostic characteristics, including peripheral small nodules, ulcer formation (“rodent ulcer”) [1], and black color (especially in Japanese [2, 3] and black people [4]). Recently, the spread of dermoscopy and accumulation of dermoscopic findings have improved the diagnostic accuracy of BCC [5]. The characteristic dermoscopic findings of BCC include arborizing vessels, spoke wheel areas, leaf-like areas, large blue-gray ovoid nests, multiple blue-gray globules, among others [6].

In this paper we experienced an interesting case that had three different features of multiple superficial types of BCCs. This present case revealed typical spoke wheel areas and leaf-like areas, and we were able to make detailed correlations between its dermoscopic and histopathologic findings.

## 2. Case Report

An 87-year-old Japanese woman had noticed small erythematous and brown-colored patches on the anterior chest, lateral neck, and lateral back for several years. She sought consultation at our hospital because she was concerned that these lesions had been gradually enlarging.

**Figure 1:**
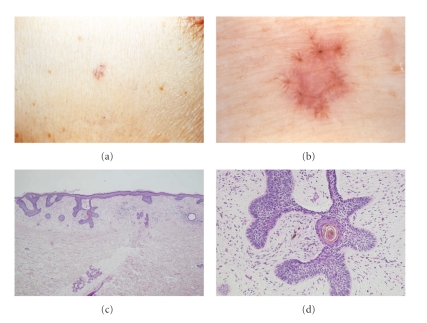
(a) The erythematous and brown-colored patch of the anterior chest. (b) Dermoscopy shows typical spoke wheel areas (pine needle-like areas). (c) Histopathology vertical spoke wheel structure is present in the center.

**Figure 2:**
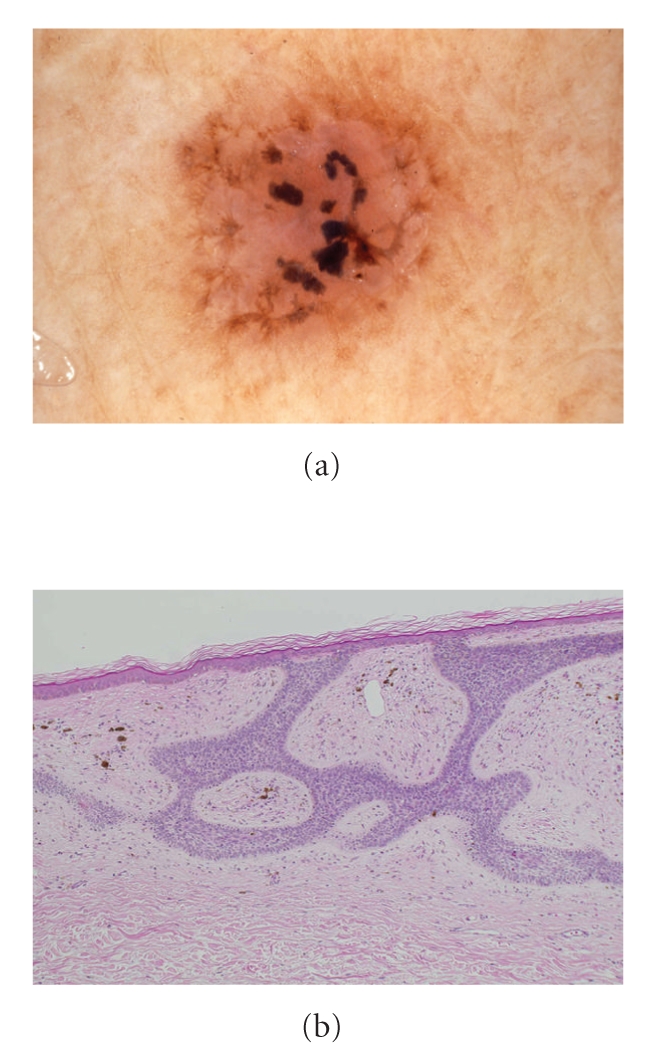
(a) Dermoscopy of the black and brown-colored patch of the lateral neck shows maple leaf-like areas. (b) Histopathologically the downward trabecular processes from the epidermis show rather tight connections in the lateral direction with other processes, and many surrounding melanophages are noted.

**Figure 3:**
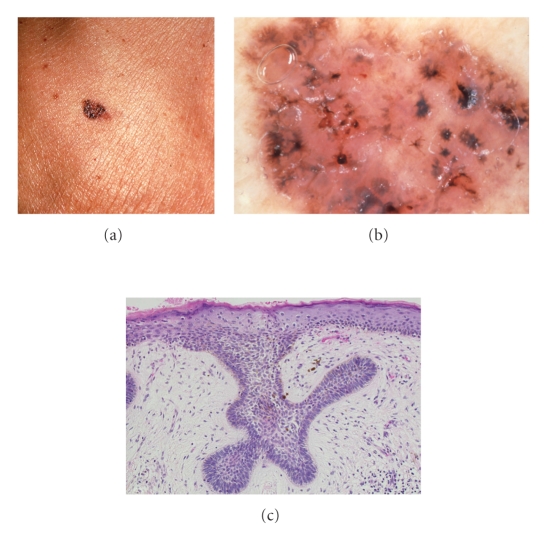
(a) The black and brown-colored patch of the lateral neck. (b) Dermoscopy shows leaf-like or petal-like areas. (c) Histopathologically, the peripheral zone of irregularly-shaped spoke wheel areas shows a strengthened brown color, and melanocytes with increased melanin pigments are observed.

Three lesions (Figures 1–3) were noted. The lesion of the anterior chest was 1.3 × 1.0 cm in size and showed an erythematous and brown-colored patch (Figure 1(a)). The lesion of the lateral neck was 1.3 × 1.3 cm in size and demonstrated a black- and brown-colored patch. The lesion of the lateral back was 1.9 × 1.5 cm in size and revealed a brown-colored patch (Figure 3(a)).

## 3. Materials and Methods

Sections from the surgically excised specimens were fixed in 10% buffered formalin and stained with hematoxylin and eosin. Dermoscopic examinations and histopathological investigations were performed, and both findings were correlated in detail.

## 4. Results

### 4.1. Dermatoscopic Findings

The erythematous and brown-colored patch of the anterior chest (Figure 1(b)): This lesion showed typical spoke wheel areas (pine needle-like structures). It had eight spoke wheel structures. Radial processes showed convergence toward a central nodule. Namely, slender processes stretched from a central axis radially. They anastomosed mutually by bands to form a polygon, which bore a close resemblance to the crystallization of snowflakes or the appearance of several skyrockets opening simultaneously during fireworks. The inner area of this plaque was pale pink in color. The areas of punctuate pigment deposit were partially obvious and partially unclear. The obvious punctuate pigments were round to oval.

The black- and brown-colored patch of the lateral neck (Figure 2(a)): in this plaque, spoke wheel structures surrounding central scar areas were present circularly, and pigmented structures were scattered in the inner side. They were partially anastomosed, which suggested a transition to the leaf-like areas. The bands connecting each structure became gradually unclear. This plaque lesion showed an amorphous black pigment deposition in the inner side and a leaf-like notch in the peripheral area. The border between the inner pink area and its surrounding normal skin was separated by brown colored belt formation. 

The black- and brown-colored patch of the lateral head (Figure 3(b)): this lesion had the same structure of Figure 2(b) basically, with the exception that it was thought to be more enlarged. The processes of the spoke wheel structures were more enlarged and dark-colored. The findings of these structures were called leak-like areas or petal-like areas. The inner potions of the petal-like areas were more whitish. Partial ulcer formation and early capillary dilatations were noted.

### 4.2. Histopathological Findings

The erythematous and brown-colored patch of the anterior chest (Figures 1(c) and 1(d)): the rete ridges were elongated and fused. The tips of the rete ridges were limited to the upper dermis; these findings were consistent with those seen in the superficial type of BCC. There were mild lymphocytic infiltrations around the nests of BCC. In addition, there was a circular or oval ring-shaped structure, which was connected with elongated rete ridge (Figure 1(c)). The ring-shaped structures had double fissures, namely, an outer black pigmented ring and an inner pigmented structure. Besides a band connecting these structures to the epidermis, there were five processes extending radially. One of those processes was connected to another process with a fine band. These processes made a spoke wheel pattern, which showed a palisading arrangement at the outermost side. These palisading nuclei were compressed to the inner side; therefore, clear cytoplasms were seen in the outer side and fissures were noted around the outer side portion. Surrounding stroma of these structures was edematous and some siderophages were observed (Figure 1(d)). 

The black- and brown-colored patch of the lateral neck: the downwardly-extending trabecular lesion from the epidermis had a rather tight connection in the lateral direction with the adjacent processes, and the number and size of melanophages had increased (Figure 2(a)). Capillaries had also proliferated, with each one showing congestion.

The black- and brown-colored patch of the lateral head: the peripheral zone of irregularly-shaped spoke wheel areas increased in their brown color, and melanocytes showed marked melanin pigments (Figure 3(c)). The surrounding area of these lesions demonstrated neither lymphocytic infiltration nor melanophages and showed rather edematous fibrosis.

### 4.3. Correlation between Dermoscopic and Histopathological Findings

The dermoscopic findings of the erythematous and brown-colored patch of the anterior chest showed a spoke wheel pattern horizontally (Figure 1(b)), and also the histopathological findings of this patch demonstrated a spoke wheel pattern vertically (Figure 1(c)). Namely, the lesion showed the spoke wheel structure both horizontally and vertically.

The dermoscopic findings of the black- and brown-colored patch of the lateral neck showed maple leaf-like areas (Figure 2(a)), and the histopathological findings of these lesions were consistent with the strengthening of the lateral connection between the processes (Figure 2(b)). Moreover, the amorphous black pigmented deposition by dermoscopy showed large proliferated melanophages and capillary proliferation with congestion histopathologically.

The dermoscopic findings of the black- and brown-colored patch of the lateral head showed irregularly-shaped spoke wheel areas with dark black color (Figure 3(b)), which was histopathologically horizontally spoke wheel structure (Figure 3(c)). Histopathologically, edematous fibrosis was present, and it was thought to be correlated with whitish areas in dermoscopy. These findings may suggest spontaneous regression of the lesion.

None of the stages of these superficial types of BCC showed any findings of either marked dilated capillary vessels (corresponding to the arborizing vessels in dermoscopy) or nodal proliferating tumor mass (consistent with blue-gray ovoid nests).

## 5. Discussion

So far, many studies have investigated basal cell carcinomas (BCCs) using dermoscopy. Recently Scalvenzi et al. described dermoscopically the characteristic features of the superficial type of BCC, correlating them with the histopathological findings [7]. However, in our review of the literature, no paper demonstrated a comparative study between horizontal spoke wheel areas by dermoscopy and vertical distinct spoke wheel structures in histopathology. In our study, we showed in detail that a 1-to-1 correlation exists between dermoscopic and histopathological findings of spoke wheel pattern and leaf-like areas. 

The dermoscopic findings of the superficial type of BCC were based on the radial structure with mutual horizontal connections. This radial structure is called spoke wheel areas or pine needle-like structures, depending on the shape. It is thought that these structures are called petal-like structures or maple leaf-like structures in accordance with the increasing width of their processes. Then, radial structures show a band-like rimming by circularly surrounding the peripheral area in the superficial type of BCC. As the lesion progresses, the rimming gradually may become unclear [5, 7], as seen in this present case. As the superficial type of BCC shows a series of these developmental processes, it finally forms a scar formation in the inner side of the lesion, together with scattered pigmented structures [2, 3]. Arborizing vessels and blue-gray ovoid nests are rather unremarkable compared to those seen in other types of BCC.

In the present case, the spoke wheel patterns, which are present in the earliest lesion of the superficial type of BCC, were recognized not only in the dermoscopic horizontal finding but also in the histopathological vertical finding. Point-like pigmented deposition in the central portion of spoke wheel areas of the superficial type of BCC in dermoscopy bore histopathologically an amorphous pigmented material with surrounding a slit formation. This slit formation fits the space filled with lubricating oil. Moreover, during the process of forming a wheel structure, nuclei were arranged at the inner side in their cytoplasm, forming a palisading pattern. This pattern resembled the normal outer root sheath structure.

## 6. Conclusions

The superficial type of BCC showed obvious spoke wheel areas or maple leaf-like areas; however, blue-gray ovoid nests or arborizing vessels were rarely noted in dermoscopy. These findings are consistent with previously reported studies [5–7]. They showed not only vertically spoke wheel structures but also horizontal spoke wheel structures.
